# Periodontal Inflammation and Dysbiosis Relate to Microbial Changes in the Gut

**DOI:** 10.3390/microorganisms12061225

**Published:** 2024-06-18

**Authors:** Angela R. Kamer, Smruti Pushalkar, Babak Hamidi, Malvin N. Janal, Vera Tang, Kumar Raghava Chowdary Annam, Leena Palomo, Deepthi Gulivindala, Lidia Glodzik, Deepak Saxena

**Affiliations:** 1 Department of Periodontology and Implant Dentistry, College of Dentistry, New York University, 345 East 24th Street, New York, NY 10010, USA; bh2138@nyu.edu (B.H.); vwt200@nyu.edu (V.T.); kra286@nyu.edu (K.R.C.A.); lp2706@nyu.edu (L.P.); deepz2311@gmail.com (D.G.); 2 Center for Genomics and Systems Biology, New York University, 12 Waverly Place, New York, NY 10003, USA; sp117@nyu.edu; 3 Department of Epidemiology and Health Promotion, College of Dentistry, New York University, 345 East 24th Street, New York, NY 10010, USA; i.m.maljanal@gmail.com; 4 Department of Radiology, Weill Cornell Medicine, Brain Health Imaging Institute Cornell University, New York, NY 10021, USA; 5 Department of Basic Sciences and Craniofacial Biology, College of Dentistry, New York University, 345 East 24th Street, New York, NY 10010, USA; ds100@nyu.edu

**Keywords:** periodontal disease, periodontal dysbiosis, periodontal inflammation, PISA, SCFA, gut dysbiosis

## Abstract

Periodontal disease (PerioD) is a chronic inflammatory disease of dysbiotic etiology. Animal models and few human data showed a relationship between oral bacteria and gut dysbiosis. However, the effect of periodontal inflammation and subgingival dysbiosis on the gut is unknown. We hypothesized that periodontal inflammation and its associated subgingival dysbiosis contribute to gut dysbiosis even in subjects free of known gut disorders. We evaluated and compared elderly subjects with Low and High periodontal inflammation (assessed by Periodontal Inflamed Surface Area (PISA)) for stool and subgingival derived bacteria (assayed by 16S rRNA sequencing). The associations between PISA/subgingival dysbiosis and gut dysbiosis and bacteria known to produce short-chain fatty acid (SCFA) were assessed. LEfSe analysis showed that, in Low PISA, species belonging to *Lactobacillus*, *Roseburia,* and *Ruminococcus* taxa and *Lactobacillus zeae* were enriched, while species belonging to *Coprococcus*, *Clostridiales*, and *Atopobium* were enriched in High PISA. Regression analyses showed that PISA associated with indicators of dysbiosis in the gut mainly reduced abundance of SCFA producing bacteria (Radj = −0.38, *p* = 0.03). Subgingival bacterial dysbiosis also associated with reduced levels of gut SCFA producing bacteria (Radj = −0.58, *p* = 0.002). These results suggest that periodontal inflammation and subgingival microbiota contribute to gut bacterial changes.

## 1. Introduction

Periodontal disease is a chronic, inflammatory condition present in more than 50% of the population [1]. It results from the interaction between subgingival dysbiotic bacteria and the host immune response [2,3,4] leading to local inflammation characterized by tissue infiltration with immune cells and high proinflammatory cytokines such as IL-8, IL-1β, IL-6, and TNFα [5] and systemic inflammation.

There is increasing evidence that the oral microbiome and periodontal inflammation play a significant role in systemic diseases, including gut disorders characterized by gut microbial dysbiosis [6,7]. The gut microbiome is the most abundant and diverse microbiome. In the human gut, there are >1000 different bacterial species [8] making up about 2 million genes (the microbiome). The gut with its microbiome is contiguous with the oral cavity, which has the second most abundant microbiome. Therefore, multiple anatomical and physiologic communications exist between the two sites [9]. In the gut, dysbiotic states are, in general, associated with a decrease in bacterial diversity (number of different bacterial species) and decrease in beneficial bacteria such as those with anti-inflammatory properties, those producing short-chain fatty acids (SCFA) such as acetate, propionate, and butyrate, or those with an intestinal-barrier-protecting effect [9,10,11]. In animal models, gut SCFA-producing bacteria have been shown to be decreased by ligature-induced periodontitis and increased by nonsurgical periodontal treatment, emphasizing the importance of periodontal inflammation in modulating gut SCFA-producing bacteria [12].

Clinical data linking periodontal disease and gut dysbiosis are shown particularly in conditions with pre-existing gut pathology [13,14,15]. In its absence, only a few small studies showed differences in the gut microbial composition between subjects with and without periodontal disease [16].

While the mechanism by which oral bacteria and inflammation can contribute to gut disorders is unknown, there is considerable evidence showing that periodontal disease can change gut bacterial composition. Periodontal-derived bacteria and inflammatory cytokines may directly reach the gut or indirectly by getting access to the systemic circulation and then reaching the gut. Recently, we have shown that clinical periodontal inflammation correlated with salivary cytokines, demonstrating a strong local oral inflammation related to periodontal inflammation [17]. Therefore, it is conceivable that clinically defined periodontal inflammation may impact gut health.

The present cross-sectional study tested the hypothesis that, in elderly subjects free of gut conditions/diseases, clinical periodontal inflammation and periodontal bacteria are directly associated with gut dysbiosis and inversely associated with gut bacteria known to produce SCFA (“healthy bacteria”).

## 2. Material and Methods

### 2.1. Study Design and Population

This is a cross-sectional study in which the subjects were recruited from an existing cohort and their characteristics were described previously [18,19]. Thirty-six (36) subjects that had both clinical periodontal measures and stool samples were included in this study. Among them, 26 subjects also had measures of subgingival microbiota. *Inclusion Criteria*: All included subjects had reported ≥12 years of education. *Exclusion Criteria*: Individuals were excluded if they had significant history or medical conditions of stroke, diabetes, uncontrolled hypertension, head trauma, any neurodegenerative disease, and chronic depression. Subjects taking anti-inflammatory medications for chronic conditions (i.e., NSAIDS and anti-TNFα) or antibiotics or having periodontal treatment within 3 months of the periodontal evaluation were also excluded. All dental exams and sample collections were standardized. Subgingival and stool sample collection and processing were conducted independently and therefore blinded from each other.

### 2.2. Dependent Variables

Primary outcomes were derived from the gut microbiota in stool samples. Using Lefse analysis [20], we identified gut bacteria at the species level that differed between the groups with high and low levels of clinical periodontal inflammation: High PISA and Low PISA. Gut pathogenic bacteria were defined as those that were abundant in High PISA, while gut beneficial bacteria were defined as those most abundant in the Low PISA group. Then, we constructed a gut dysbiotic index (Gut-DI) defined as the ratio of the mean of gut pathogenic bacteria to the mean of beneficial bacteria. This approach is modeled after other indexes published in the literature [21,22].

Secondary outcomes: Short-chain fatty acid (SCFA) bacterial index was derived by the cumulative mean of each of butyrate, propionate, and acetate bacterial indexes in the gut microbiome. These SCFAs are most commonly associated with health benefits [23,24]. All these indexes were derived by averaging bacterial abundances that are known to produce the respective SCFA as summarized by Akhadar [25]. Therefore, butyrate bacterial index was derived from the following bacterial abundances: *Ruminococcus_bromii*, *Anaerostipes_s*, *Coprococcus_eutactus*, *Roseburia_s*, and *Faecalibacterium prausnitzii*. Propionate bacterial index was derived from the following bacterial abundances: *Akkermansia_muciniphila*, *Ruminococcus_Other_A*, *Bacteroides_s*, *Coprococcus_eutactus*, *Roseburia_s*, and *Dialister_s* and acetate bacterial index was formed from *Bifidobacterium_s*, *Bacteroides_s*, *Streptococcus_s*, *Clostridium_s*, *Blautia_s*, *Ruminococcus_s*, and *Akkermansia muciniphila.*

### 2.3. Independent Variables

Independent variables (main exposure) were the clinical measures of periodontal disease: periodontal inflamed surface area (PISA) scores. PISA scores were calculated from periodontal pocket depth (PD) and bleeding on probing (BOP) using the formula from Nesse’s publication (http://www.parsprototo.info/ (accessed on 5 June 2024)) [26] accessed February, 2024. PISA scores [26] were dichotomized into High (pathogenic) vs. Low PISA (nonpathogenic) groups as we previously described using 450 mm^2^ as the threshold [17]. Based on this cut-off, 12 subjects were High PISA and 24 subjects were Low PISA.

The secondary independent variable (exposure) was the subgingival dysbiotic index (Subgingival-DI), as published previously by us and others [19]. It was defined as the abundance ratio at the genus level of bacteria associated with periodontal disease (*Treponema*, *Porphyromonas*, and *Tannerella*) to healthy bacteria (*Rothia* and *Corynebacterium)* [19]. Higher numbers indicate a less healthy oral microbiome.

### 2.4. Clinical Evaluations and Sample Collection

Periodontal exam: Subjects received an oral-periodontal examination, as previously described [17]. Briefly, this exam encompassed examination of 6 surfaces of each tooth for probing depth, clinical attachment loss (CAL), and bleeding on probing (BOP). Pocket depth measures were assessed at six sites per tooth using a Michigan probe and defined as the linear distance in millimeters from the gingival margin to the base of the periodontal pocket in millimeters. Bleeding on probing (BOP) was assessed at each probing site after the quadrant probing. Demographic (age, gender, and education), systemic factors (co-morbidities), oral (brushing, dentist visits, and prophylaxis), and social (smoking) measures were obtained by a standardized examiner-conducted interview at the time of the oral examination [18]. Smoking was defined as never smoking vs. current/past smoking. Brushing was classified as brushing once/day vs. >once/day. Prophylaxis was defined by having cleanings each 3 months or at intervals > 3 months.

Subgingival sample collection: Subgingival bacterial samples were collected from the four deepest periodontal pockets, as previously described [27]. The samples were pooled into one vial and stored at −80 °C.

Stool was collected as previously published [28]. Subjects were provided with a stool collection kit containing detailed written instructions, a collecting hat, a stool collecting kit (ALPCO), and gel ice packs. Stool was collected at home and brought to the NYUCD at the appointment within 24 h. Stool processing and storage was standardized and samples were stored at −80 °C until sequencing.

### 2.5. Microbiome Assessment and Analyses

#### 16S rRNA Amplification and Sequencing

We used the 16SrRNA methodology as previously published in this study [29,30]. Briefly, DNA was extracted from the subgingival plaque and stool samples. Using PCR, the V3–V4 region of 16S rRNA gene was amplified, sequenced, and the reads were clustered in operational taxonomic units (OTU) identifying bacterial ranking. We report our analyses at species levels. Alpha diversity was assessed for the observed OTUs and Shannon Diversity Index.

### 2.6. Statistical Methods

Statistical analyses were performed using IBM SPSS (v27, IBM Corp., Armonk, NY, USA). Continuous data are presented as means and standard deviation (SD) and categorical data as percentages. To evaluate biomarker group differences for continuous variables, *t*-test and Mann–Whitney U (MWU) test were used, whichever was appropriate. For categorical variables, Chi-Square tests were used. Normality was tested by Kolmogorov–Smirnov and log10 transformation was used to normalize the distributions for each microbial index. For microbiome analyses, we used the linear discriminant effect size analysis (LEfSe). LEfSe uses an algorithm that combines statistical with biological significance to reveal biomarker clusters [20]. Effect size (LDA = linear discriminative analysis scores expressed in log_10_) provides an estimation of the magnitude of the observed effect. The effect size (LDA) to estimate the magnitude of the observed effect was computed using the default settings, *p* < 0.05 and LDA ≥ 2 [20].

### 2.7. Statistical Analysis

Association between Gut-DI and SCFA bacterial indexes and PISA and Subgingival-DI were evaluated using multiple regression analyses adjusted for age. In initial models, we evaluated the association of potential confounders with the bacterial indexes: gender, education, BMI, behaviors (smoking, brushing, and dentist visits), systemic conditions (0 vs. 1 medical conditions). None of these were significant and, therefore, not included in the final models. Age was also not significant; however, due to its reported association with both gut bacterial dysbiosis [31,32] and PISA/Subgingival-DI, it was included in all final models. Given the exploratory nature of this study, an unadjusted *p* < 0.05 level of significance was used.

## 3. Results

The characteristics of our population are shown in Table 1. Our population was relatively homogeneous. Most were white, elderly, and highly educated. Females were more represented. Subjects were relatively healthy with >30% not reporting any medical conditions, while 60% reported ≤ 1 medical condition. Two subjects were current smokers. PISA scores were similar as a function of age, gender, education, BMI, smoking, or the number of systemic conditions. All subjects had CAL ≥ 5 mm and, therefore, they had stage III and IV periodontitis.

Subjects (n = 36) that were included because they provided the information needed to complete these analyses were similar to those not included (n = 40) on measures of age (*p* = 0.91), years of education (*p* = 0.36), BMI (*p* = 0.65), PISA (*p* = 0.76), number of teeth (*p* = 0.80), and Perio staging diagnosis (*p* = 0.55). Thus, these subjects appear representative of the larger group. There were no differences in High and Low PISA in gender (*p* = 0.45), race (*p* = 0.71), smoking (*p* = 0.45), brushing (*p* = 0.55), prophy frequency (*p* = 0.55), or the presence of ≤1 medical condition (*p* = 0.81).

There were no differences in the gut alpha diversity assessed by observed and Shannon indexes between PISA+ and PISA− groups (*p* = 0.12 and *p* = 0.80).

### 3.1. Gut Pathogenic and Beneficial Bacteria Were Differentially Enriched in High/Low PISA

**Figure 1:**
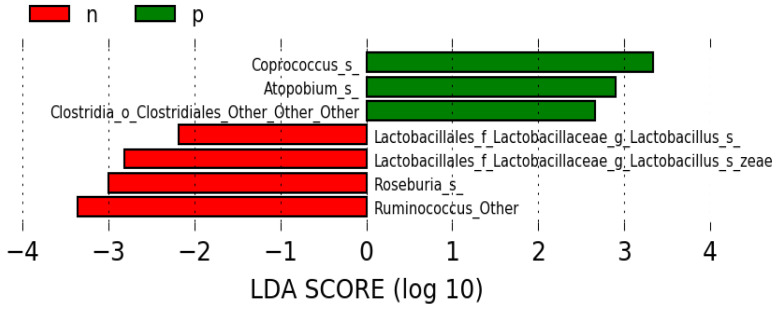
Differences in gut bacterial composition between pathogenic (high PISA-p) and nonpathogenic (low PISA-n). Using LEfSe, we determined the most abundant gut bacteria in the 12 High PISA (p) and the 24 Low PISA (n) groups at species level. Of importance, gut bacteria enriched in Low PISA are known as gut beneficial bacteria, while the bacteria associated with the High PISA are linked to gut pathology.

Using LEfSe, we determined the most discriminative features between the 12 High and the 24 Low PISA groups at the species level. As shown in Figure 1, the species from taxa *Coprococcus*, *Atopobium,* and *Clostridiales* were abundant in High PISA, while *Lactobacillus zeae*, and species from genera *Lactobacillus*, *Roseburia*, and *Ruminococcus* were abundant in the Low PISA (red) group.

### 3.2. Gut-DI Correlates with Firmicutes-to-Bacteroidetes Ratio 

**Figure 2:**
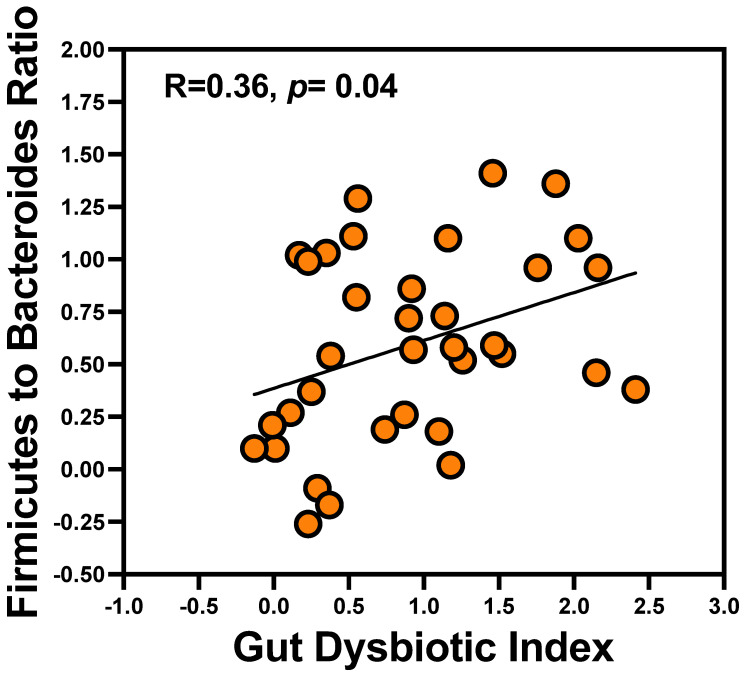
Gut dysbiotic index correlates with Firmicutes-to-Bacteroides ratio. Although there is no consensus definition for gut dysbiosis [21], Firmicutes/Bacteroidetes (F/B) ratio is accepted as an important index of gut dysbiosis [33]. We showed that Gut-DI defined in our study correlated with Firmicutes-to-Bacteroides ratio (R = 0.36, *p* = 0.04).

Although there are dissenters [21], Firmicutes/Bacteroidetes (F/B) ratio is accepted as an important index signaling pathogenic intestinal changes/gut dysbiosis [33]. Therefore, we tested whether Gut-DI correlated with F/B ratio. As shown in Figure 2, there was a direct correlation between the Gut-DI constructed using Lefse and F/B ratio with R = 0.36, *p* = 0.04.

### 3.3. Gut-DI Inversely Associated with Gut SCFA Bacterial Index

**Figure 3:**
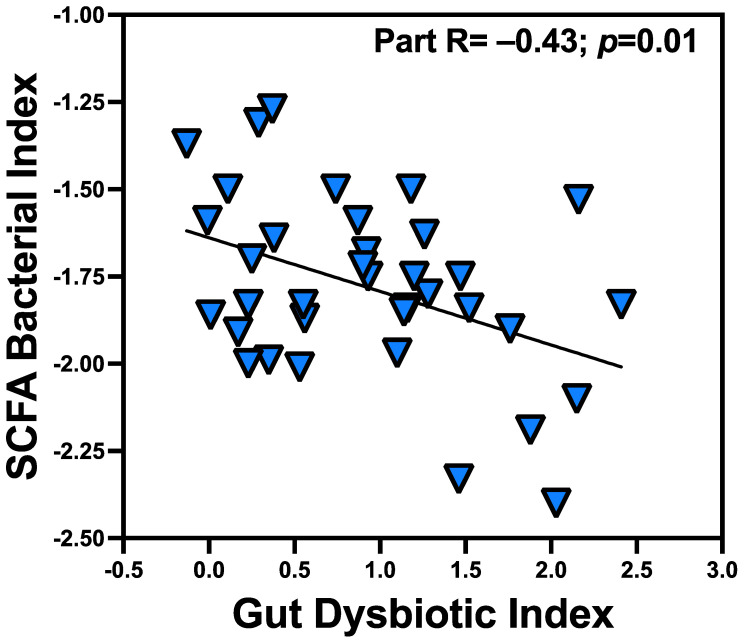
Gut-DI inversely associated with gut SCFA bacterial index. In regression analyses, the gut SCFA bacterial index inversely correlated with Gut-DI (Radj = −0.43, *p* = 0.01).

A growing body of evidence suggests that some bacteria are particularly beneficial through SCFA production [25,34]. Therefore, we sought to determine if Gut-DI relates to SCFA-producing bacteria. In regression analyses, the gut SCFA bacterial index was predicted by Gut-DI (Radj = −0.43, *p* = 0.01); Figure 3 shows the relationship between the Gut-DI and SCFA bacterial index: as Gut-DI increased, the abundances of gut SCFA bacteria decreased. Among the three components of the gut SCFA bacterial indexes, the propionate (Radj = −0.34, *p* = 0.044) and butyrate (Radj = −0.55, *p* = 0.001) significantly associated with Gut-DI, while the acetate index was approaching significance (Radj = −0.29, *p* = 0.091).

### 3.4. PISA Inversely Associated with Gut SCFA Bacterial Index

**Figure 4:**
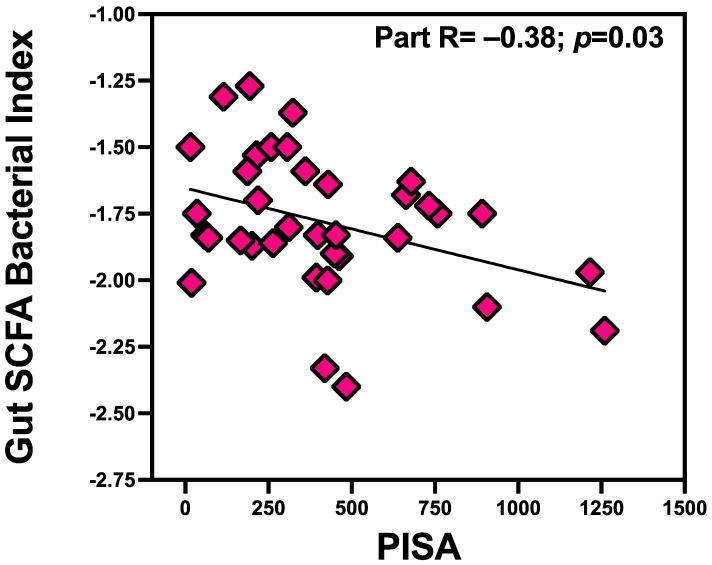
PISA inversely associated with gut SCFA bacterial index. In regression analyses, PISA score inversely associated with gut SCFA bacterial index (Radj = −0.38, *p* = 0.03).

We tested the hypothesis that PISA will also impact the SCFA-producing bacteria. In regression analyses, the gut SCFA bacterial index was predicted by PISA score (Radj = −0.38, *p* = 0.03). Figure 4 shows the relationship between PISA and the SCFA bacterial index: as PISA scores increased, the abundances of SCFA-producing bacteria decreased. Among the three SCFA-producing bacteria, PISA associated significantly with the propionate (Radj = −0.42, *p* = 0.01) and acetate (Radj = −0.34, *p* = 0.045) but not with butyrate-producing bacteria (Radj = −0.14, *p* = 0.41).

### 3.5. Subgingival-DI Inversely Associated with Gut SCFA Bacterial Index

**Figure 5:**
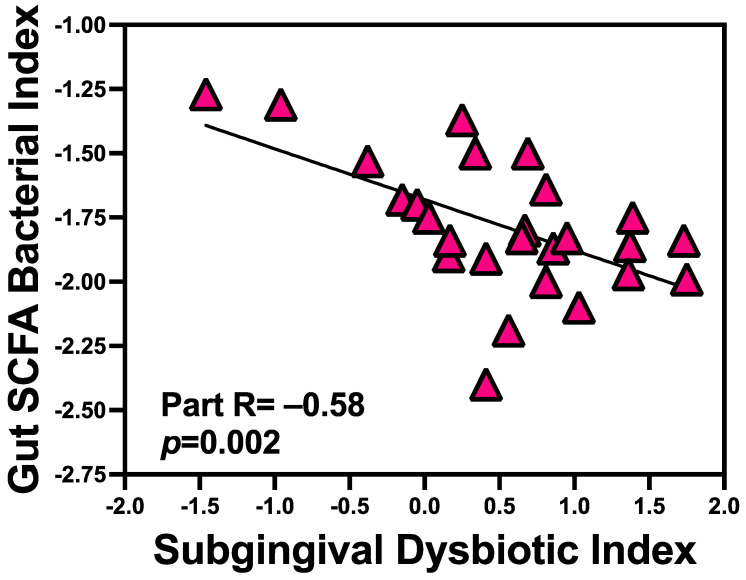
Subgingival-DI inversely associated with gut SCFA bacterial index. In regression analyses, subgingival-DI inversely correlated with gut SCFA bacterial index (Radj = −0.58, *p* = 0.002).

We hypothesized that subgingival bacteria will also impact the SCFA-producing bacteria. In regression analyses, the gut SCFA bacterial index was predicted by Subgingival-DI (Radj = −0.58, *p* = 0.002). Figure 5 shows the inverse relationship between the subgingival dysbiotic index and gut SCFA bacterial index: as subgingival dysbiotic index increased, the abundances of SCFA bacteria decreased. Among the three components of the gut SCFA bacterial indexes, the propionate and acetate bacterial indexes significantly associated with the subgingival dysbiotic index (propionate index: Radj = −0.56, *p* = 0.002; acetate index: Radj = −0.53, *p* = 0.007), while butyrate did not (Radj = −0.28, *p* = 0.22). Subgingival-DI failed to correlate with Gut-DI (Radj = 0.14, *p* = 0.52).

## 4. Discussion

### 4.1. Main Findings

In elderly subjects, free of gut disorders, clinical periodontal inflammation was associated with indicators of dysbiosis in the gut. These changes were specifically related to reduced activity in healthy gut bacteria that produce SCFA. Subgingival bacterial dysbiosis was also associated with reduced levels of gut SCFA bacteria. These results suggest that periodontal inflammation and subgingival microbiota contribute to gut dysbiosis and are consistent with animal models showing modulation of gut dysbiosis by induction and treatment of periodontal disease [12].

### 4.2. Clinical Periodontal Inflammation and Gut Dysbiosis

We tested the hypothesis that clinical periodontal inflammation affects gut bacterial changes. We found that, in subjects with high clinical periodontal inflammation, gut microbiota were characterized by enrichment mainly in bacteria associated with gut pathology, while, in those with low clinical periodontal inflammation, the gut bacteria were enriched in beneficial bacteria. The gut pathogenic bacteria enriched in High PISA such as *Clostridia* [35] and *Atopobium* [36] lead to gut inflammation, increase permeability, translocation of bacteria and lipopolysaccharides (LPS) to the systemic circulation [37], and consequent systemic inflammation [38,39,40]. *Coprococcus* species, in contrast, have been associated with health benefits due to their butyrate production. We can speculate that their enrichment in High PISA may be due to specific *coprococcus* species/subspecies that may have pathogenic capabilities or their enrichment is a reaction to the presence of a high dysbiotic environment. Low PISA was enriched in beneficial bacteria whose effects are contrary to the pathogenic bacteria. For example, *Lactobacillus_s_* and *Lactobacillus zeae* are known for maintaining IBD remission [41]. Species from Lactobacillaceae family produce lactic acid as the final product of glucose fermentation, with immune health and pathogenic bacteria inhibitory effects [41]. In fact, *Lactobacillus* is considered probiotic due to its benefits, safety profile, and its production of acetate [42]. Other beneficial bacteria such as *Rosburia* and *Ruminococcus* are known for SCFA production (see below).

Clinical periodontal inflammation is a characteristic of periodontal disease and can be assessed by PISA scores using current measures of periodontal disease: PD and BOP [26,43]. PISA has been found to associate with plasma CRP [44], a marker of systemic inflammation and several systemic conditions [45,46,47], and correlates with salivary cytokines [17]. In addition to inflammation, periodontal disease is characterized by periodontal tissue destruction as a pathognomonic feature. Periodontal disease expression depends on the interaction between periodontal bacteria and host immune response. It affects more than 50% of people over 50 [3,4].

There are no studies directly linking clinical periodontal inflammation (assessed by PISA) to the gut. However, studies showed the importance of periodontal disease in gut pathology and is supported by periodontal animal models demonstrating gut dysbiosis, oral bacterial translocation to the gut, gut inflammation (increased CRP, Th1, and Th17 cytokines), increased permeability, systemic inflammation, and distant pathology such as AD pathology [48,49,50]. Ectopic colonization of oral bacteria in the intestine leads to TH1 cell induction and inflammation [51]. Transplanting saliva from patients with severe periodontitis into mice changed gut microbiota composition with higher abundance of *Porphyromonadaceae* and *Fusobacterium* and lower *Akkermansia* compared to controls (no perio) [13]. Reduced periodontal inflammation in animal models reduced gut infiltration with immune cells and production of inflammatory cells, particularly Th1- and Th17-related cytokines [52].

The gut microbiota is highly complex and there is significant variation among individuals. Therefore, to date, there is no gold standards to define healthy gut microbiota or gut dysbiosis [53]. However, several studies defined a change in the gut Firmicutes-to-Bacteroides ratio as a gut imbalance that was linked to obesity [54,55], metabolic syndrome [56], or autism [57]. We find that our Gut-DI correlated with Firmicutes-to-Bacteroides ratio, suggesting that our Gut-DI reflects gut dysbiosis at higher ranking levels.

We hypothesized that clinical periodontal inflammation would also change the abundance of selective gut SCFA-producing bacteria. The inverse relationship between Gut-DI and SCFA bacterial index is not surprising. While dysbiotic index serves as a surrogate for bacterial dysbiosis, dysbiosis or normobiosis are defined by the composition and functional make-up of the whole bacterial community and not by just a few bacteria composing the dysbiotic index. Therefore, as expected, as dysbiotic index increased, the beneficial bacteria decreased.

Beneficial microbiota produce bioactive molecules such as short-chain fatty acids (SCFA) with anti-inflammatory properties, immune-regulating activity, intestinal barrier protection [25,58], and reduce systemic inflammation [59,60,61]. The main SCFAs produced by colonic anaerobic bacteria are acetic acid, propionic acid, and butyric acid, which represent 90% to 95% of the SCFAs, with a proportion of about 60%, 20%, and 20%, respectively [62,63]. Therefore, our SCFA bacterial index was composed of these three SCFA-producing bacteria. However, studies determining the bacterial index with SCFA determination are desirable and important to perform.

### 4.3. Subgingival Dysbiosis and Gut Dysbiosis

Our data showed that subgingival dysbiosis inversely associated with gut SCFA bacterial index, further supporting the hypotheses that periodontal disease associated with clinical periodontal inflammation and periodontal bacteria associated with gut microbial changes.

Over 200 bacterial species colonize the subgingival biofilm and, among them, several are enriched in periodontal disease (i.e., *Porphyromonas gingivalis (PG)* and *Treponema denticola*), while others (i.e., *Rothia* and *Corynebacterium*) are enriched in periodontically healthy subjects [64]. One recent study found that subgingival dysbiotic index defined as a ratio of *Treponema*, *Porphyromonas*, and *Tannerella* to healthy bacteria (*Rothia* and *Corynebacterium*) [19] associated with periodontal disease and PISA. We used this index in a previous study to show correlations with a systemic effect [19].

The mechanism by which periodontal-disease-associated inflammation/dysbiosis may induce gut dysbiosis is unknown. However, we can speculate that oral bacteria and associated inflammatory molecules directly or indirectly can affect the gut bacterial composition. Approximately ~1 L to 1.5 L of saliva containing ~10^11^ oral bacterial cells daily reaches the intestinal tract. While many oral bacteria are destroyed, evidence showed that a significant number reach the gut [65], stimulate the immune system, and, with predisposing influences, can cause pathology [51]. The indirect mechanism implies that bacteria and inflammatory cytokines obtain access to the systemic circulation and then reach the gut or influence gut response.

We published previously that two salivary indexes composed of six cytokines correlated with PISA [17]. In the present study, salivary cytokine indexes failed to correlate with gut measures and thus failed to support the direct effect of oral cytokines on the gut.

**Figure 6:**
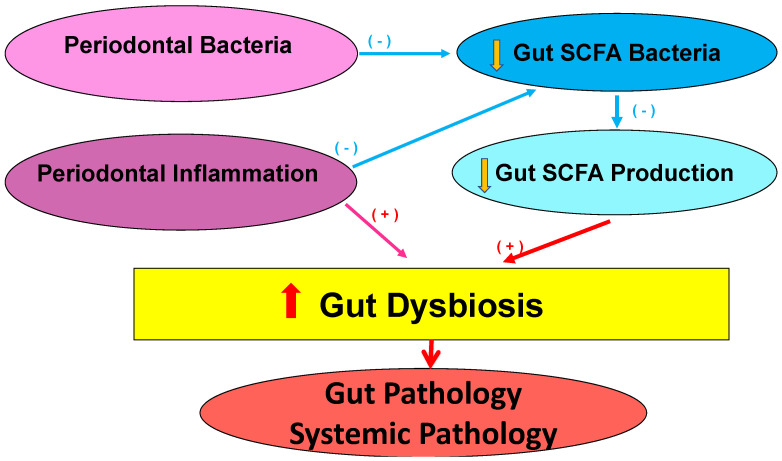
Model of hypothetical pathways from periodontal inflammation and dysbiosis to gut bacterial changes. Periodontal bacteria (subgingival pathogenic bacteria) contribute to reductions in the SCFA-producing bacteria and, therefore, the amount of SCFA. Reduced SCFA are known to contribute to gut dysbiosis, with negative consequences on the gut. Periodontal inflammation independently or interactively regulates gut SCFA in addition to contributing to gut dysbiosis. Gut dysbiosis further contributes to other systemic diseases. (blue arrows = negative regulators; red arrows = positive regulators.

Another possible mechanism is via oral bacteria. It is possible that periodontal bacteria reach the gut directly. Clinical data linking periodontal disease and gut dysbiosis are shown particularly in conditions with pre-existing gut pathology [14,15,66,67,68,69]. We tested whether Subgingival-DI associated with Gut-DI. While, in this small sample, Subgingival-DI failed to correlate with Gut-DI, in regression analyses, Subgingival-DI associated with gut SCFA bacterial index, suggesting that the subgingival periodontal bacteria effect may be on SCFA-producing bacteria. It is possible that SCFA-producing bacteria are more sensitive to subgingival dysbiosis or the study is too small to detect these changes. Gut-DI correlated with SCFA bacterial index, suggesting that SCFA may drive gut dysbiosis. Therefore, we propose a model (Figure 6) in which A. subgingival pathogenic bacteria induce reductions in the gut SCFA-producing bacteria and, therefore, the amount of gut SCFA. Lower gut SCFA, in turn, regulate gut dysbiosis with negative consequences on the gut. B. Periodontal inflammation independently or interactively regulates gut SCFA-producing bacteria in addition to contributing to gut dysbiosis. These results are consistent with other studies. In an animal model, ligature-induced periodontitis-induced gut dysbiosis, intestinal pathology, and changes in bacterial function. Moreover, 4 weeks after, a nonsurgical periodontal treatment partially restored the gut microbiota toward health. Consistent with our study, periodontal treatment increased bacteria with SCFA-producing effects, again suggesting that subgingival bacteria effects are targeted towards SCFA bacteria [12]. This model should be tested in larger, longitudinal studies.

Our subjects were free of any inflammatory bowel disease or any other gut disorders. Nevertheless, subjects with high clinical periodontal inflammation had gut bacterial changes. The clinical significance of these gut bacterial changes is unknown. It is possible that the subjects’ gut condition is mild, occult and undetected, or unreported. It is also possible that the magnitude of the gut bacterial changes was not severe enough or not accompanied by predisposing factors to manifest as a gut clinical condition. In the absence of a gut clinical condition, the gut dysbiosis may have deleterious systemic effects. Gut dysbiosis is considered a major trigger of systemic inflammation both in animal models and in humans and gut dysbiosis has also been linked to several inflammatory conditions such as obesity [54], metabolic syndrome [70], diabetes [71], cardio-vascular disease [72], and AD [73,74]. And still another possibility is that our stool sampling may be an early indicator of gut dysbiosis.

### 4.4. Strengths and Weaknesses

Several strengths characterized our study. Our sample was quite homogeneous, consisting of elderly, well-educated, and relatively healthy individuals. None had gut disorders. Periodontal measures and subgingival and stool collections were standardized and the microbiome assessments were determined blindly to the periodontal assessment.

There are several limitations related to our study that include the design, population characteristics, and sample size. As a cross-sectional study, it shows only a correlation of periodontal inflammation/subgingival bacteria to gut measures and the direction of the association cannot be determined. It is also possible that gut microbiota could affect periodontal inflammation and subgingival dysbiosis [75]. The number of subjects was limited. All subjects had stage III and IV periodontitis. Therefore, these results may not apply to the general population. It is to be noticed that subjects included and not included in the study did not differ in their demographic characteristics. An additional limitation is lack of information about the dietary factors that can change the gut microbiome.

Stool sampling was used as an index of gut microbiota and there are limitations in using the stool as an index of gut microbiome. Stool represents the colon microbiota rather than the entire section of the gut [76] and studies found a good representation of the luminal gut [77]. In addition, stool sampling is the most established method of characterizing the gut microbiome and studies have shown significant relationships between the stool microbiome and gut/systemic conditions [78].

In conclusion, in this study, we found that PISA scores significantly associated with gut dysbiotic index and these associations were independent of age. Our results support our hypothesis that clinical periodontal inflammation can be used as a correlate of the severity of gut bacterial changes. Our results also showed that PISA and subgingival periodontal bacteria associated with gut SCFA bacterial index, suggesting that oral inflammation/subgingival periodontal bacteria may affect the production of SCFA.

Larger longitudinal studies assessing periodontal-disease-associated inflammation/bacteria as well as SCFA production would be desirable. However, interventional studies using periodontal treatment as the intervention and gut bacterial changes and SCFA production as outcomes would not only validate our results but would also point towards mechanistic pathways. Treatment of periodontal disease is aimed at reducing bacterial dysbiosis and local/systemic inflammation. This treatment would be expected to prevent changes in the gut bacteria and SCFA. There are several periodontal treatment options, including scaling and root planing, local, systemic antibiotics, antiseptics [79,80], and surgical procedures. Antibiotics/antiseptics could have a direct effect on the gut bacteria and, therefore, these treatments are not recommended when investigating the effect of oral inflammation/dysbiosis on the gut bacteria. Scaling and root planing and supportive periodontal disease therapy with rigorous home care are the gold standard for periodontal disease therapy [43]. With this treatment, the supra- and subgingival bacterial biofilm enriched in pathogenic bacteria, bacterial products, and calculus deposits are removed, thereby inducing a subgingival environment characterized by health-associated bacteria [81,82] and reduced local and systemic inflammation. This is a relatively inexpensive, noninvasive procedure and would constitute a model for a drug-free treatment. Its effects on the gut would be due to reduced periodontal inflammation and dysbiosis.

## Figures and Tables

**Table 1:** Characteristics of the study population by PISA groups.

	Total	Low PISA	High PISA	*p*-Value
N	N = 36	N = 24	N = 12	
Demographics				
Age (Mean (SD))	66.7 (9.1)	66.08 (9.5)	67.8 (8.5)	0.59
Education (Mean (SD))	17.1 (2.3)	16.9 (2.4)	17.4 (2.2)	0.51
Gender n (%)				
Female	24 (66.7)	17 (70.8)	7 (54.5)	0.45
Race n (%)				
White	32 (88.9)	21 (87.5)	11 (91.7)	0.71
Medical Health				
BMI	26.9 (6.2)	26.8 (6.1)	27.1 (6.7)	0.92
Medical Cond (N (%))				
1 or 0	22 (61.1)	15 (62.5)	7 (58.3)	
>1	14 (38.9)	9 (37.5)	5 (41.7)	0.81
Behavior				
Smoking (%)				
Yes	12 (33.3)	9 (37.5)	3 (25.0)	0.45
Oral health behavior				
Brushing (%)				
Once/day	7 (19.4)	4 (16.7)	3 (25.0)	
>1/day	29 (80.6)	20 (83.3)	9 (75)	0.55
Prophy				
Each 3 months	7 (19.4)	4 (16.7)	3 (25)	
>q3months	29 (80.6)	20 (83.3)	9 (75)	0.55
MW = Mann–Whitney U Test				

## Data Availability

The original contributions presented in the study are included in the article. Further inquiries can be directed to the corresponding author.
